# Molecular Detection of Bladder Cancer by Fluorescence Microsatellite Analysis and an Automated Genetic Analyzing System

**DOI:** 10.1100/tsw.2007.176

**Published:** 2007-09-17

**Authors:** Sarel Halachmi, Michal Cohen, Raymond Szargel, Nadin Cohen

**Affiliations:** ^1^ The Department of Urology Rambam Medical Center, Haifa, Israel; ^2^ The Department of Genetics, Tamkin Human Molecular Genetics Research Facility, Faculty of Medicine, Technion Israeli Institute of Technology, Haifa, Israel

**Keywords:** cancer, bladder cancer, urology, oncology

## Abstract

To investigate the ability of an automated fluorescent analyzing system to detect microsatellite alterations, in patients with bladder cancer. We investigated 11 with pathology proven bladder Transitional Cell Carcinoma (TCC) for microsatellite alterations in blood, urine, and tumor biopsies. DNA was prepared by standard methods from blood, urine and resected tumor specimens, and was used for microsatellite analysis. After the primers were fluorescent labeled, amplification of the DNA was performed with PCR. The PCR products were placed into the automated genetic analyser (ABI Prism 310, Perkin Elmer, USA) and were subjected to fluorescent scanning with argon ion laser beams. The fluorescent signal intensity measured by the genetic analyzer measured the product size in terms of base pairs. We found loss of heterozygocity (LOH) or microsatellite alterations (a loss or gain of nucleotides, which alter the original normal locus size) in all the patients by using fluorescent microsatellite analysis and an automated analyzing system. In each case the genetic changes found in urine samples were identical to those found in the resected tumor sample. The studies demonstrated the ability to detect bladder tumor non-invasively by fluorescent microsatellite analysis of urine samples. Our study supports the worldwide trend for the search of non-invasive methods to detect bladder cancer. We have overcome major obstacles that prevented the clinical use of an experimental system. With our new tested system microsatellite analysis can be done cheaper, faster, easier and with higher scientific accuracy.

## INTRODUCTION

Transitional cell carcinoma (TCC) of the bladder is one of the most common malignancies in the Western world[1,2]. This tumor occurs in two different forms, a chronic and slowly progressing superficial disease, and muscle invasive disease[3]. Superficial tumors are amenable for local excision however even after complete transurethral resection of these tumors, up to 70% of the patients will experience tumor recurrence and up to 25% may progress to a higher stage or grade[4]. Due to this unique malignant behavior the main goal in the management of bladder cancer is early detection of relapsing tumors. Currently, cytological examination of voided urine sample is the only accepted non-invasive method for detection of bladder cancer[5]. The major obstacle of this technique is the low sensitivity (less than 50%) in patients with low-grade tumors[5,6,7,8]. In order to overcome the low sensitivity of urine cytology analysis newer molecular techniques, such as the use of microsatellite analysis, have been developed as a non-invasive tool for detection of bladder cancer. Despite highest sensitivity and specificity major limitation of this method were, radioactivity, time consuming and subjective analysis of data[9-23]. The aim of our study was to investigate whether microsatellite analysis for the detection of bladder cancer could be facilitated by an automated quantitative analyzing genetic system.

## METHODS

Eleven patients with recurrent TCC who were scheduled for transurethral resection of their bladder tumors (TURBT) were prospectively enrolled in our study. Prior to tumor resection, a fresh urine and blood sample were taken from the patient. Urine, blood and the resected tumor sample from each patient were subjected for microsatellite analysis. The clinicopathological data of these patients is summarized in Table 1.

**TABLE 1**
**Clinicopathological and Genetic Data**

| *Patient* | *Sex/Age* | *PT Stage* | *pT Grade* | *Cytology* | *LOH* | *Shift* |
|---|---|---|---|---|---|---|
| 1 | M/74 | T4 | II | + | | FGA |
| 2 | M/78 | Ta | II | - | D18S51 | |
| 3 | F/72 | T3b | II-III | + | D17S654<br>D16S476<br>D9S162 | |
| 4 | M/64 | T4 | II-III | + | D9S747 | |
| 5 | F/75 | Ta-T1 | III | Atypia | D20S48 | D17S695 |
| 6 | M/82 | Ta | II-III | - | D17S695 | D21S1245 |
| 7 | M/91 | Ta | I-II | - | D17S695<br>D9S171 | |
| 8 | M/56 | Ta | II-III | - | D21S1245 | D17S695<br>D9S171 |
| 9 | M/72 | T1 | II | - | D16S476 | D9S162 |
| 10 | F/61 | Ta | I-II | - | D21S1245 | |
| 11 | M/89 | Ta | II | Atypia | D20S48 | |

### Sample Collection and DNA Isolation

Prior to surgery fresh urine and blood samples were collected from each patient. Blood was collected by venipuncture into an EDTA containing tube for normal control DNA. Urine samples were immediately precipitated in 2000 RPM for 10 minutes, the upper fluid was discarded and sediment was used for DNA extraction. Fresh tumor tissue was frozen immediately in liquid nitrogen and microdissected as previously described by Boyle et al[24] DNA extraction from lymphocytes, urine sediment and tumor samples was done by digestion in SDS-PK at $48^0$c overnight, followed by phenol/chloroform extraction and ethanol DNA precipitation, as previously described by Van der Riet et al.[25]

## PCR Amplification, and Automatic DNA Fragments Size Measurement Microsatellite Analysis

DNA extracted from tumor, urine and lymphocytes was subjected or microsatellite analysis using a panel of 20 markers. Those microsatellite markers has already been selected by preliminary studies at the Johns Hopkins Hospital which were found to be highly specific and sensitive for the detection of bladder cancer[21,22]. The primers (Genset, research genetics inc., Paris, France) were fluorescently labeled and then subjected to PCR amplification. Genomic DNA was subjected to 1 PCR cycle at a denaturing temperature of 95$^o$C for 4 minutes, followed by 35 PCR cycles of 30 seconds in 95$^o$C, 30 seconds of varying annealing temperatures ranging from 54-58$^o$C according to the primer specifications, an extension step at 72$^o$C for 30 seconds and a final additional extension step at 72$^o$C for 6 minutes on the PCR programmable thermal controller (MJ Research Incorporation, USA).

The PCR products were diluted to a ratio of 1:30, and 1μl was transferred to ependorf tubes containing 14 μl of denaturing solution (tamra formamide). The tubes were put into the automated genetic analyser (ABI Prism 310, Perkin Elmer, USA) and were subjected to fluorescent scanning after radiation with argon ion laser beams. The fluorescent signal intensity measured by the genetic analyzer determined the PCR product size in terms of base pairs. Analysis of the matched blood, tumor and urine samples allowed us to search for genetic alterations such as LOH or instability (SHIFT = gain or loss of nucleotides) within the tumor and urine DNA that was ultimately compared to that of the normal blood sample (which served as our control).

## RESULTS

Patient's clinical and pathological data is presented in table 1. Using our 20 microsatellite testing marker panel[21,22], we found LOH or microsatellite alterations in 11 out of 11 patients (100%). In each case, the microsatellite alterations found in urine samples were identical the tumoral alterations.

In three patients urine cytology was positive for malignant cells and an additional 2 atypical cells were found. The 6 remaining cytology's were negative. Ten patients had loss of heterozygocity 8 and one alteration was found. Five patients had single locus genetic aberrations and in the remaining 6 more than one site was involved.

The use of the genetic analyzing unit allowed us to automate the analysis and to use florescent, instead of radioactive labeling. We were able to analyze all samples using 4 different labeling in one run (i.e 11 samples X 4 different microsatellite primers = 44 samples). Scanning results are quantitative and displayed in analog and graphic forms preventing non-quantitative subjective inaccurate reading.

## DISCUSSION

Assessment of the genetic changes in human tumors and body fluids at multiple chromosomal sights using microsatellite analysis has already been described and validated in various human neoplastic processes including bladder cancer[9,10,14-22]. The detection of genetic alterations in human malignancies has implications concerning diagnosis, prognosis, follow-up and therapeutic decisions in malignant disease. Microsatellite analysis has proved to be a sensitive and specific non-invasive technique in the detection of malignant neoplasms. Primary tumors harbor genetic alterations such as microsatellite alterations or chromosomal deletions at suppressor gene loci, which become manifested as LOH. Due to the fact that bladder cancer is considered a clonal tumor (it arises from the uncontrolled spread of a single transformed cell[26]), microsatellite analysis is a highly suitable technique for the screening and follow-up of patients with bladder cancer. In our series we detected 11 out 11 (100%) patients with bladder cancer by fluorescent microsatellite analysis of matched blood, tumor and urine samples. In the original work by Mao et al,[21] a set of 13 primers was used. Steiner et al[22] added seven more primers to the original panel in order to increase the sensitivity. The addition of primers in order to analyze additional loci will certainly increase the sensitivity of this method, but would not be technically feasible to be performed by a non-research laboratory. Our method enabled us to test 3 matched samples from one patient in 30 different loci during a single working session. The increase in panel markers increases the sensitivity without an increase in the cost, effort or labor due to the use of an automated system.

Our main goal in this study was to automate microsatellite analysis and improve product reading and analysis ability and to eliminate the work with radioactive substance. Using fluorescent labeling we could eliminate the hazard of radioactivity and imply the use of the genetic analyser. This device is an automated unit that is capable of determining the size and quantity of DNA fragments. The ABI Prism 310 was able to reduce human labor and the time needed to run and read the results. We have also overcome a major limitation of the oldest radioactive analysis, which is its qualitative nature. In the oldest system the intensity of the labeled products and the separation between two highly close molecular size PCR products is judged qualitatively only by the naked eye. Using the genetic analyser we were able to measure quatitatively the exact intensity of the fluorescent radiation and the exact product size. In this way the experimental results could be evaluated according to a mathematical scale and not just by subjective personal impression, which might not be reproducible in some difficult to analyze cases.

We were able to eliminate the risk of radioactivity, reduce the need for human labor, and facilitate the time needed to obtain results for clinical decisions. In addition, the ability to quantify the results allows for a more accurate analysis. Our clinical results validate and confirm the reliability of microsatellite analysis of cells that are exfoliated in the urine. The sensitivity of the procedure was consistent with earlier reports that demonstrated a high detection rate of malignant DNA in urine samples. Furthermore, the specificity of our method was identical to that reported by the Mao et al and Steiner et al[21,22].

In conclusion, we demonstrate the ability to detect bladder tumor non-invasively by fluorescent microsatellite analysis of urine samples. Our study supports the worldwide trend for the search of non-invasive methods to detect bladder cancer. We have overcome major obstacles that prevented the clinical use of an experimental system. Further studies needed to determine the exact sensitivity and specificity of microsatellite analysis on a large-scale population are still warranted. However, with our new tested system it can be done cheaper, faster, easier and with higher scientific accuracy.

**Thi.s article should be cited as follows:**

Halachmi, S., Cohen, M., Szargel, R., and Cohen, N. (2007) Molecular detection of bladder cancer by fluorescence microsatellite analysis and an automated genetic analyzing system. *TheScientificWorldJOURNAL:* TSW Holistic Health & Medicine 7, 1553–1557. DOI 10.1100/tsw.2007.176.
